# When Should the Emphasis on Schistosomiasis Control Move to Elimination?

**DOI:** 10.3390/tropicalmed3030085

**Published:** 2018-08-15

**Authors:** W. Evan Secor, Daniel G. Colley

**Affiliations:** 1 Division of Parasitic Diseases and Malaria, Centers for Disease Control and Prevention, Atlanta, GA 30329, USA; was4@cdc.gov; 2 Center for Tropical and Emerging Global Diseases and Department of Microbiology, University of Georgia, Athens, GA 30602, USA

**Keywords:** schistosomiasis, control, elimination, Africa, operational research, goals, guidelines

## Abstract

The stated goal of the World Health Organization’s program on schistosomiasis is paraphrased as follows: to control morbidity and eliminate transmission where feasible. Switching from a goal of controlling morbidity to interrupting transmission may well be currently feasible in some countries in the Caribbean, some areas in South America, northern Africa, and selected endemic areas in sub-Saharan Africa where there have been improvements in sanitation and access to clean water. However, in most of sub-Saharan Africa, where programmatic interventions still consist solely of annual mass drug administration, such a switch in strategies remains premature. There is a continued need for operational research on how best to reduce transmission to a point where interruption of transmission may be achievable. The level of infection at which it is feasible to transition from control to elimination must also be defined. In parallel, there is also a need to develop and evaluate approaches for achieving and validating elimination. There are currently neither evidence-based methods nor tools for breaking transmission or verifying that it has been accomplished. The basis for these statements stems from numerous studies that will be reviewed and summarized in this article; many, but not all of which were undertaken as part of SCORE, the Schistosomiasis Consortium for Operational Research and Evaluation.

## 1. Introduction

In 2001, the World Health Assembly (WHA) passed resolution 54.19 that called for regular administration of praziquantel for schistosomiasis (and albendazole or mebendazole for soil-transmitted helminthiasis, STH) to at least 75% of school-aged children at risk of morbidity [1]. The passage of WHA 54.19 helped stimulate governments, pharmaceutical companies, and private donors to provide the resources to allow a considerable expansion of mass drug administration (MDA) for schistosomiasis and STH along with the operational research to inform programs on how to best deliver treatments and monitor impact. Buoyed by this progress, as well as by promising advances towards the elimination of other neglected tropical diseases (NTDs), the WHA passed resolution 65.21 in 2012, which called for interrupting the transmission of schistosomiasis (elimination) where appropriate [2].

The exuberance that led to WHA 65.21 was based on progress towards the WHA 54.19 goals and elimination as a public health problem (<5% prevalence of high-intensity infections in children), success with other NTD elimination programs, and the desire to set program endpoints. However, the critical modifier ‘where appropriate’ is often overlooked in discussions of schistosomiasis elimination and currently remains undefined. This has led to a certain degree of confusion for programs and dissonance among schistosomiasis stakeholders and the larger NTD community in the development of strategic plans and the allocation of resources.

Schistosomiasis control programs in most endemic countries still consist almost solely of MDA with praziquantel. Considerably less progress has been made towards WHA 54.19’s other goals of promoting access to safe water, sanitation, and health education. WHA 65.21 also called for guidance to help countries determine when to switch from control to elimination campaigns, how to implement them, and the process for documenting success. However, these requested guidelines still have not been generated, primarily because the data needed to generate them (i.e., from successful elimination programs) simply do not exist. Thus, one of the most pressing schistosomiasis research needs is to define when it is appropriate to switch from a focus on control to a goal of interrupting transmission.

The few countries that have successfully interrupted the transmission of schistosomiasis have largely been successful when their public health programs were combined with social and economic development. Even for these countries, the process to certify elimination remains unclear. In this review, we detail why we believe that for now an emphasis on interrupting transmission in most of sub-Saharan Africa is overly ambitious. There is no question that elimination remains the laudable and ultimate goal, but, for the immediate future, efforts should be focused on laying the groundwork that will be necessary to accomplish it [3]. Interventions beyond MDA must become widely available, and the evidence regarding when to pursue and how to document elimination must be generated. Clearly, operational research and the development and evaluation of the essential tools needed for elimination and verification should move forward, but we propose that a reasonable balance needs to be struck between the emphases placed upon control and elimination efforts.

## 2. Once-Annual MDA of School-Aged Children Is Not Enough to Interrupt the Transmission of Schistosomiasis

MDA alone has led to substantial progress towards elimination of several NTDs. For example, MDA for lymphatic filariasis stops or nearly stops transmission, and annual MDA can continue until adult worms die with little risk of new infection. However, with schistosomiasis, given the rapid rates of reinfection that can occur in endemic areas, the incomplete effectiveness of praziquantel, and the considerable amplification of parasite numbers that occurs within the intermediate snail host, MDA alone is unlikely to achieve elimination. Furthermore, if interventions are stopped before bringing prevalence down to the still undefined level at which transmission cannot be sustained, rapid recrudescence is likely.

The Schistosomiasis Consortium for Operational Research and Evaluation (SCORE; https://score.uga.edu/) is an initiative funded by the Bill and Melinda Gates Foundation that has the goal to provide schistosomiasis control program managers the information they need to better do their jobs. The bulk of SCORE-funded operational research has focused on studies to compare different MDA strategies. In western Kenya, when children in primary schools with an initial *Schistosoma mansoni* prevalence of 10–24% were provided school-based treatment (SBT) at high coverage (>90%), one round of treatment was sufficient to drastically reduce prevalence and intensity of infection. However, subsequent rounds, even when provided annually over four years, never brought infection levels below approximately 50% of the baseline prevalence [4]. Thus, even with rigorous implementation in a research setting, MDA alone was not adequate for accomplishing elimination.

## 3. Modifications to Improve MDA Effectiveness Still Do Not Achieve Elimination

It is possible that community-wide treatment (CWT) in the villages with 10–24% baseline prevalence could have been more effective to achieve the interruption of transmission, especially if there are some adults (e.g., fishermen, sand harvesters, shepherds, or car washers) who have occupations that make them important contributors to transmission in a particular setting. However, in keeping with the current World Health Organization (WHO) guidelines that only recommend CWT when prevalence is greater than 50%, the SCORE studies in areas with 10–24% initial prevalence did not evaluate the impact of CWT. Nevertheless, a SCORE study on Zanzibar (both Pemba and Unguja), which was designed to understand how to achieve elimination in an area that had already received extensive MDA for control of *Schistosoma haematobium* infection for many years, did evaluate CWT [5,6]. This study was part of a major effort called ZEST (Zanzibar Elimination of Schistosomiasis Transmission) involving multiple partners, including a strong commitment by political leaders, to achieve elimination throughout Zanzibar. The SCORE study within ZEST examined three strategies designed to drive schistosomiasis haematobia to elimination. All three strategies utilized twice yearly CWT, either alone, in combination with a community-designed behavioral change intervention, or alongside periodic focal snail control with niclosamide. In areas with low baseline prevalence, it is clear that while schistosomiasis prevalence and infection intensity decreased in most shehias (villages), in some it did not [7]. This variability in response to MDA, resulting in ‘persistent hotspots,’ has been observed in other SCORE studies [8,9], as well as other tropical disease control programs [10,11]. Persisting hotspots are a reality that must be appreciated, identified, and addressed when contemplating elimination goals. Whether the goal is morbidity control or elimination, both field studies and mathematical modeling suggest that it will be necessary to adapt MDA approaches to address persistent hotspots, including monitoring MDA programs more often than the currently recommended 5–6 years [12,13].

## 4. Interventions in Addition to MDA Are Needed for Elimination but Are Challenging to Apply

The development and implementation of a community-designed behavioral intervention on Zanzibar required both considerable time and effort [14]. The interventions that the community developed under the guidance of a social scientist and a trained team resulted in the greatly increased involvement of children in activity-based health education about schistosomiasis and the use of participant-designed and -installed concrete washing platforms and clean water to wash clothes. While enabling communities to identify their own problems and solutions is attractive with respect to both self-determination and sustainability, the financial and time investments required are an obstacle to many large control programs.

Adding snail control to MDA also takes some time to be fully implemented due to the training required and the need to identify water-contact sites likely to be important in the transmission of schistosomiasis. On Zanzibar, snail control was implemented focally in identified water-contact sites in which snails were found [5]. This generally occurred 3–4 times per year. Because of the seasonality of the rainy seasons, the sites needing treatment with niclosamide varied at each visit. Thus, as with behavioral change interventions, mollusciciding needs to be tailored to individual sites for effective use and requires considerable training and person-power.

There was a time when snail control was the mainstay of schistosomiasis control. In Africa, the Danish Bilharziasis Laboratory (DBL) and other institutions trained many field personnel in the science and art of snail control. Both molluscicides and environmental interventions designed by water and sanitation engineers were effective snail control measures. In East Asia, both the Chinese and Japanese programs were highly proficient in this practice, and it was effective for reducing schistosomiasis prevalence in humans [3]. However, upon the advent of a safe, generally effective drug, funding for these approaches dwindled to a point where they are now largely non-existent in control programs. It is encouraging that two training workshops for snail control were held in 2017 under the auspices of the WHO/AFRO’s Expanded Special Project for the Elimination of NTDs (ESPEN), one on Zanzibar and the other in Burkina Faso, and the WHO has now produced a snail control manual [15]. Nevertheless, the malacology capacity in most of Africa remains very limited, and most ministries have not invested in snail control for their schistosomiasis control programs. This contrasts with programs for vector-borne NTDs, where measures to control mosquitoes, blackflies, tsetse flies, and the like are an integral part of control and elimination efforts. Encouragingly, at least a few environmental engineers are beginning to think about transmission and control of schistosomiasis [16]. Getting more people trained in these areas and getting more NTD programs to acknowledge and utilize these approaches is very likely to be essential for effective elimination within an acceptable timeframe.

As for many other public health issues, the availability of water, sanitation, and hygiene (WASH) in addition to MDA is likely the key intervention for breaking schistosomiasis transmission. For example, schistosomiasis used to be a severe problem in several islands in the Caribbean. In the 1970s and 1980s, Saint Lucia had many residents who suffered from severe hepatosplenic disease as a result of *S. mansoni* infection [17]. After the Rockefeller/Ministry of Health Research and Control project ended in 1981, there was no specific control program for schistosomiasis. However, the economic development that has occurred over the last 30 years has resulted in a vast expansion of WASH throughout most of the island and a concurrent reduction, and possible interruption of transmission, of schistosomiasis. While it may not be possible to completely attribute this change to the greater utilization of WASH (competitor snails were introduced in some areas as part of Research and Control project and some have flourished at the expense of *Biomphalaria glabrata*), the coincident decline in schistosomiasis prevalence is striking. Puerto Rico and the Dominican Republic have similarly experienced great economic development leading to increased WASH and reductions in schistosomiasis prevalence to the point where they are ready for surveys to determine whether elimination has been achieved. The late Professor George Hillyer, who was a leading schistosomiasis researcher in Puerto Rico from the 1970s until his death in 2015, liked to quip that the best control measure for schistosomiasis was concrete. Considering that Puerto Rico at one time had levels of schistosomiasis that rivaled anywhere in Africa and is now ready to verify elimination, even in the absence of large-scale MDA, his point seems prescient.

It is likely that elimination will only be achieved in countries (or areas within countries) with some basal level of a clean water supply and sanitation. However, what that basal level is remains unknown, and there is a clear need for operational research to determine what that level might be. Preliminary results from a follow-on SCORE study in western Kenya designed to identify the factors associated with schistosomiasis persistent hotspots suggest that villages with higher levels of sanitation have greater reductions in prevalence following MDA compared with those that do not [18]. Research to define what level of WASH is needed for effective schistosomiasis control or elimination will require input from behavioral scientists and water and sanitation engineers. Unfortunately, people who have this training and also know or care about schistosomiasis control are in short supply. Nevertheless, schistosomiasis investigators and control advocates, including the WHO, have now begun to cultivate and develop partnerships within the WASH sector [19]. These partnerships need to grow and lead to real WASH implementation to accomplish the sought after goals of both schistosomiasis control and its elimination.

## 5. What Else Is Needed for Elimination?

Another challenge to achieving elimination is the difficulty of demonstrating significant decreases in infection levels in areas with very low baseline prevalence and intensity. If there is any appreciable variability, such as due to persistent hotspots, demonstrating statistically significant reductions from 2 to 0.5% prevalence requires the evaluation of very large numbers of individuals, which would likely exceed current budgets or implementation abilities of most programs. Proving that prevalence is 0% will require the development of reliable sampling schemes and the wide availability of sensitive tools. Urine filtration is not adequate for monitoring low levels of *S. haematobium* infection, as was made clear when urine specimens collected in Zanzibar were assayed by the highly sensitive and specific up-converting phosphor-circulating anodic antigen (UCP-CAA) assay. Parallel use of these two assays on the same urine specimens yielded considerably higher prevalence estimates with the UCP-CAA assay in most shehias [20]. Schools thought to have low prevalence by urine filtration (mean = 3.4%) had an average prevalence of 17.2% by UCP-CAA assay. These differences could not be explained as false positive results, as latent class analysis estimated that the UCP-CAA assay had 90.1% specificity and 97.0% sensitivity [20]. To implement and validate elimination, surveillance tools more sensitive than parasitologic microscopy will be needed. These tools will also need to be sufficiently cost effective for use on large numbers of individuals and perhaps amenable to pooling schemes [21]. At this time, the UCP-CAA assay is only available in a few specialized laboratories, and the costs are too high for routine use in control programs.

The need for a tool appropriate for validating elimination is shared by efforts to eliminate *S. mansoni*. As with *S. haematobium*, microscopy-based assays fail to detect many light *S. mansoni* infections. SCORE studies in Burundi demonstrated that greater than 85% of schools with 0% prevalence based on stool examination tested positive for schistosomiasis when the commercially available point-of-care/circulating cathodic antigen (POC-CCA) test was used [22]. Thus, many areas that appear to be approaching elimination as measured by parasitologic methods may in fact still have a high prevalence of low intensity schistosomiasis. Unfortunately, the POC-CCA assay is not without certain limitations of its own. In areas that have control programs that have achieved very low levels of prevalence and intensity, such as Egypt, or areas that once had schistosomiasis but appear to have eliminated *S. mansoni* transmission, the use of the POC-CCA assay indicates approximately 5–10% trace positive results. Some of these can be attributable to very, very low-level infections, but many are likely false positives [23]. Thus, the POC-CCA is not an adequate tool for validating elimination of transmission, and additional work is needed to define the sampling strategies, tests, and results to confirm that transmission has been interrupted [24].

Testing for schistosome-specific antibodies may also have some utility. Because individuals remain antibody positive to many if not most schistosome antigens long after they have been cured of infection, the currently available serologic tests are only useful in areas where transmission was likely interrupted a number of years before (e.g., the Caribbean) and when persons younger than the projected transmission interruption date demonstrate negative serology. Serologic tests based on crude antigen mixtures, as well as many purified native antigens, do not distinguish between active and former infections. Work to identify recombinant antigens to which the antibody response is more rapidly lost is ongoing, but as of now, antibody testing is only useful for demonstrating elimination that has occurred in the past, not programs that are actively trying to interrupt transmission.

Through the work with the more sensitive antigen detection assays, another challenge has become apparent. Although praziquantel is still indispensable for MDA programs and thankfully, no widespread clinical resistance has developed, it may not be nearly as effective at killing schistosomes as previously thought. In SCORE studies in western Kenya where children with schistosomiasis were treated with praziquantel and retested with the POC-CCA assay, only about 50% cleared infection after one round of treatment, compared with the 70–90% clearance rates observed when parasitologic methods are used to calculate efficacy [25]. Standard doses of praziquantel are even less effective in very young children [26], which is a further cause for concern even as pediatric formulations of praziquantel are undergoing clinical trials (https://www.pediatricpraziquantelconsortium.org/). As with snail control, much of the investment in identifying additional drugs to treat schistosomiasis disappeared with the availability of praziquantel. There is no mistaking that it is still the most readily available and important component of schistosomiasis control throughout the world, but it is becoming clear that additional research on compounds used either alone or in combination with praziquantel should be pursued in order to realize and sustain elimination, or in the event that stable resistance to praziquantel were to arise in schistosome populations.

A final need is a clear definition of when elimination has been achieved. Single serosurveys of children in Morocco, Saint Lucia, and the Dominican Republic have found no individuals with a positive antibody response. However, a single evaluation is unlikely to provide convincing evidence that transmission has been interrupted. But, how many surveys and of what age groups would suffice? Is it possible to only survey in areas of a country that have historically had schistosomiasis transmission, or is it also necessary to evaluate areas that have never had documented infections? If so, is the same stringency of surveillance needed in both areas? Can demonstration of high-level WASH usage or the absence of appropriate intermediate host snails or xenomonitoring be used to bolster claims that transmission has been interrupted? Is absence of infection in schoolchildren sufficient to prove elimination, or must every adult who may have been infected decades in the past be identified and cured before elimination can be certified? Because there are so few places where transmission has been interrupted and fewer still where it has been verified, developing the schistosomiasis elimination guidelines has the feel of building the airplane while learning to fly it. Modeling of data from field studies in elimination projects, both those that are successful as well as those that are not, will be important to answer these questions.

## 6. New Challenges

In addition to the obstacles to interrupting transmission detailed above, other recent developments that have come to light from schistosomiasis field studies further complicate the goal of achieving elimination. First, rather than the list of countries endemic for schistosomiasis shrinking in recent years, new foci of schistosomiasis have recently been identified in Corsica and Myanmar [27,28]. How these sites were established is unclear; however, human seeding of intermediate host snails that were already present suggests a role for increased global migration and tourism in establishing new transmission foci [29]. Another factor that may impede elimination efforts is the possible contribution of non-human hosts in maintaining the life cycle. While the zoonotic contribution to *Schistosoma japonica* transmission is widely recognized, its role in maintaining environmental life cycles of *S. mansoni* and *S. haematobium* is less clear [30]. Propagation of the life cycle of these species in reservoir hosts and snails could hinder efforts to interrupt transmission or reinitiate transmission in humans following completion of elimination programs [31]. The identification of hybrid human and animal schistosomes in West Africa further complicates this possibility as hybrid species could possibly have a wider host range and greater vigor, thereby increasing transmission potential through the infection of multiple definitive hosts [32].

## 7. What Is the Way Forward?

While there are settings where elimination of schistosomiasis transmission have been achieved [33], what is usually not acknowledged is how rarely elimination efforts have succeeded and that this has only occurred in relatively isolated situations. It is also often not appreciated that thus far, elimination has always required both extensive public health measures coupled with infrastructure development and a sustained investment over an extended period of time. In the case of Japan, it required the many decades-long use of a multi-pronged major public health push against helminth diseases [34], and then, as the fight was gaining real traction in terms of control, it was coupled with social and economic development that translated into broad-based water supply and sanitation. Even so, for at least 15 years after the elimination of schistosomiasis japonica, the government of Japan maintained a surveillance system for infected snails and people [35].

We and others maintain that elimination of schistosomiasis in sub-Saharan Africa is an unachievable goal using school-based praziquantel MDA alone, and a better use of current resources there would result in more effective control of schistosomiasis morbidity [36]. Interruption of transmission will only be possible with the introduction of additional control measures. Nevertheless, as this part of the world continues to develop, the schistosomiasis community needs to be planning for the future. By this, we mean thinking ahead about what will be needed to achieve and maintain elimination. Again, Japan provides an example. Anyone who has been so fortunate to have visited Japanese gardens in places like Kyoto, Nara, or Nikko knows that the lovely and tranquil scenes one enjoys, such as through a gracefully arched tree across a lily-dappled pond, did not ‘just happen’. It took foresight and work long ahead of when you enjoyed that idyllic botanical scenery as art. That is how schistosomiasis elimination in sub-Saharan Africa should be considered in 2018. There is a need to determine what tools will be needed and how to use them to accomplish and verify elimination and a need to plan how adequate surveillance will be accomplished. Efforts to develop these tools and plans should begin now, not at the expense of much needed control efforts, but in addition to them and alongside them. A haiku at the entrance of such a garden could read as follows:

Seasons of much work

Elimination your goal

Control on your way.

## 8. Conclusions

Given the obstacles to elimination that we describe, we propose that the major emphasis for schistosomiasis programs in sub-Saharan Africa should still be control of morbidity. Achieving this goal will require better definitions of morbidity due to schistosomiasis and the development of adaptive strategies that are based on more frequent program monitoring and go beyond standard school-based annual MDA. At the same time, we also propose that to be ready to move to a goal of elimination requires a commitment to investments in the basic and operational research needed to develop the tools and strategies that will be essential to achieve and verify elimination. Waiting to do so until countries are ready to move to elimination would be shortsighted and result in continued, long-term control efforts or, in their absence, the inevitable transmission rebound that has been observed following many historical and more recent control efforts.
